# Retraction Note: Senescence impairs direct conversion of human somatic cells to neurons

**DOI:** 10.1038/s41467-022-33635-9

**Published:** 2022-10-17

**Authors:** Chong-kui Sun, Di Zhou, Zhen Zhang, Liming He, Fan Zhang, Xiaowei Wang, Jie Yuan, Qianming Chen, Ling-Gang Wu, Qin Yang

**Affiliations:** 1 Cancer Biology Division, Washington University School of Medicine, Saint Louis, Missouri 63108 USA; 2 State Key Laboratory of Oral Diseases, Sichuan University, Chengdu, 610041 China; 3 National Institute of Neurological Disorders and Stroke, Bethesda, Maryland 20892 USA; 4 Medical College, Jinan University, Guangzhou, 510632 China

**Retraction to**: *Nature Communications* 10.1038/ncomms5112, published online 17 June 2014

The editors have retracted this article. An internal investigation by the Washington University Committee on Research Integrity has been unable to verify the integrity of data in Fig. 1c, Fig. 4a, Fig. 4b. No adequately documented raw data is available to support the result presented in the article. In addition, the images in Fig. 6c are a cropped and rotated version of the shp19 panel of Fig. 5c. Ling Gang Wu and Zhen Zhang agree to retract the Article. Liming He, Chong-kui Sun, Di Zhou, Fan Zhang, Xiaowei Wang, Jie Yuan, Qianming Chen and Qin Yang did not reply or could not be contacted.

